# Editorial: The agency of educational artifacts: reimagining the role of robots in cognitive development

**DOI:** 10.3389/frobt.2025.1760597

**Published:** 2026-04-01

**Authors:** Maria Impedovo

**Affiliations:** Department of Education, Aix-Marseille Université, Marseille, France

**Keywords:** agency, artifact, cognitive, education, robotics

## Introduction

In contemporary education, the tools we provide to learners do far more than present content—they shape how learners think, interact, and grow. Over decades, educational artifacts have evolved from simple mechanical devices to digital tools, and now to robots endowed with cognitive, emotional, and social capacities. The launch of this Research Topic — *“The Agency of Educational Artifacts: Reimagining the Role of Robots in Cognitive Development”* — calls us to examine this evolution. We invited contributions exploring how robotics and AI-infused tools can transform educational practices, augment human learning, and create new dynamics in classrooms and beyond. The four published articles in this Research Topic converge on a shared vision: to explore non-anthropomorphic and modular robotic systems as educational artifacts—not passive tools, but agents capable of scaffolding cognition, supporting social inclusion, and fostering creative problem solving. Collectively, they offer evidence that robots can transcend their traditional role as mere instruments and become active partners in learning.

## From tangible cubes to modular hands: broadening the spectrum of educational robots

One of the cornerstones of this Research Topic is the emphasis on embodied, modular, non-anthropomorphic robots—systems that may not look human, but whose physical form and affordances invite new kinds of interaction and learning. In *Comparative analysis of creative problem solving tasks across age groups using modular cube robotics*, participants of different age groups completed creative problem-solving tasks using modular cubes (Anik and Romero). This study reveals how manipulable robotic artifacts can mediate cognitive development and creative thinking—extending beyond traditional, screen-based learning. Likewise, *Play robots to develop competences* explores playful robotic systems as vehicles for competence development (Panelli et al.). It argues that robotics can—and should—go hand-in-hand with play: a core pillar of human learning, especially in early development. This article presents a structured framework to design robot-mediated play that is inclusive, goal-oriented, and developmentally beneficial. These works remind us that educational robots do not need to mimic humans to matter—their value lies in their affordances, in embodied presence and manipulability: tangible objects that children (or learners of any age) can handle, rebuild, adapt, test—and thereby learn through doing.

## Engineering mastery: robots to support technical and STEM learning

Robotic artifacts are not only suited for early childhood play or creativity tasks, but also for higher-level technical education. In *Refined active learning to reliably grasp Control Systems and Mechatronics at undergraduate level via a robotic hand*, a robotic hand is used as a teaching aid to help undergraduates grasp control systems and mechatronics (Zavala-Yoé et al.). This underscores the potential of robotics to ground abstract technical concepts in physical, manipulable form—making complex ideas tangible and thus easier to understand. In doing so, it expands the reach of educational robotics from childhood and play contexts to formal technical education, bridging the gap between theoretical knowledge and real-world, hands-on understanding. The robot becomes a cognitive artifact: a mediator between abstraction and action, enabling students to experiment, fail, adjust, and ultimately internalize complex engineering concepts.

## Inclusion and pedagogy: robots as agents of social and educational equity

Perhaps the most socially relevant contribution comes from *Educational robotics as a strategy for social inclusion and pedagogical intervention in vulnerable youth communities* (Acosta-Amaya et al.). In this research, robotic tools are deployed not merely as educational toys or STEM aids, but as part of an inclusive pedagogical intervention targeting vulnerable youth communities.

This study highlights robotics’ potential as a lever for social inclusion: by offering accessible, engaging, and scaffolded learning opportunities, educational robots can help overcome inequities in access to quality education, cognitive stimulation, and social participation. In contexts where traditional schooling may struggle, robotic artifacts—given their versatility—can become powerful agents for empowerment.

## Toward a new paradigm: educational artifacts as agents, not tools

What unites these diverse contributions is a conceptual shift: from viewing robots as mere tools, to seeing them as agents—artifacts with agency that can mediate, scaffold, and shape learning. In doing so, they challenge the traditional human-centered model of education, where humans design, instruct, and evaluate, and tools are passive. Instead, robots become co-actors, embedded in learning environments, influencing cognitive trajectories, social dynamics, and educational outcomes.

This paradigm resonates with a broader movement in cognitive science and robotics toward embodied cognition—the view that cognition emerges through dynamic interaction between agent and environment, not solely within the brain. Implementing this perspective in education could herald a transformation: classrooms no longer as passive information-transfer venues, but as living, interactive ecosystems where humans and robotic artifacts co-construct learning paths.

## Challenges and future directions

The integration of robots as educational agents also surfaces a series of conceptual, pedagogical, and ethical challenges that warrant careful consideration. First, questions of design and affordances remain central. Determining which robotic morphologies, physical affordances, and interaction modalities most effectively support diverse learning processes—from early playful exploration to advanced technical reasoning—is essential for aligning technological capabilities with pedagogical intent. Second, issues of scaffolding and instructional design arise. Educators must negotiate how robotic artifacts are embedded within curricular sequences and how they mediate learning activities. This includes balancing learner autonomy with structured guidance, and ensuring that robotic interventions complement—rather than substitute for—human pedagogical judgment. Third, the increasing presence of robotics in educational settings foregrounds concerns related to ethics, equity, and access. Ensuring that all learners benefit from robotic technologies requires attention to affordability, inclusivity, and the risks of technological determinism. Moreover, safeguarding learners’ socio-emotional wellbeing and maintaining meaningful human–robot relational boundaries remain critical considerations.

Finally, questions concerning long-term impacts and evaluation persist. While short-term learning gains are often documented, the sustained cognitive, social, and developmental effects of prolonged engagement with robotic agents are still largely unknown. Addressing this gap necessitates robust longitudinal research designs as well as cross-cultural studies capable of capturing variations across social, economic, and pedagogical contexts. Together, these challenges underscore the need for a research agenda that is interdisciplinary, methodologically diverse, and attentive to the broader ecological conditions in which educational robotics operates.

## Conclusion

The four papers in this Research Topic together paint a compelling picture: educational robotics is no longer a novelty reserved for after-school clubs or tech-savvy early adopters. Rather, robotics—especially embodied, modular, non-anthropomorphic systems—has the potential to become a core component of future educational ecosystems: tools that act, respond, scaffold, and grow with learners. By situating robots as cognitive and social agents—not just programmable machines—we open the door to richer, more inclusive, more flexible educational practices. Such a shift not only broadens what we understand by “educational artifact,” but also reimagines what education itself can be: a collaborative dance between humans, materials, and machines; a space where creativity, inclusion, and mastery all find room to flourish. It is our hope—as a Topic Editor—that this Research Topic will inspire future research along these lines, and that the educational robotics community will continue to explore, critically and creatively, the agency of artifacts in human development.

